# COVID-19 Vaccines for HIV-Infected Patients

**DOI:** 10.3390/v13101890

**Published:** 2021-09-22

**Authors:** Maria M. Plummer, Charles S. Pavia

**Affiliations:** 1 Department of Clinical Specialties, Division of Pathology, New York Institute of Technology, NYIT College of Osteopathic Medicine, Old Westbury, NY 11568, USA; mplummer@nyit.edu; 2 Department of Biomedical Sciences, New York Institute of Technology, NYIT College of Osteopathic Medicine, Old Westbury, NY 11568, USA; 3 Division of Infectious Diseases, New York Medical College, Valhalla, NY 10595, USA

**Keywords:** HIV, AIDS, SARS-Cov-2, COVID-19, SARS-Cov-2 vaccines, mRNA

## Abstract

Nearly 40 years have passed since the initial cases of infection with the human mmunodeficiency virus (HIV) were identified as a new disease entity and the cause of acquired immunodeficiency disease (AIDS). This virus, unlike any other, is capable of causing severe suppression of our adaptive immune defense mechanisms by directly infecting and destroying helper T cells leading to increased susceptibility to a wide variety of microbial pathogens, especially those considered to be intracellular or opportunistic. After T cells are infected, HIV reproduces itself via a somewhat unique mechanism involving various metabolic steps, which includes the use of a reverse transcriptase enzyme that enables the viral RNA to produce copies of its complementary DNA. Subsequent physiologic steps lead to the production of new virus progeny and the eventual death of the invaded T cell. Fortunately, both serologic and molecular tests (such as PCR) can be used to confirm the diagnosis of an HIV infection. In the wake of the current COVID-19 pandemic, it appears that people living with HIV/AIDS are equally or slightly more susceptible to the etiologic agent, SARS-CoV-2, than the general population having intact immune systems, but they may have more serious outcomes. Limited clinical trials have also shown that the currently available COVID-19 vaccines are both safe and effective in affording protection to HIV/AIDS patients. In this review, we further explore the unique dynamic of HIV/AIDS in the context of the worldwide COVID-19 pandemic and the implementation of vaccines as a protective measure against COVID-19, as well as what immune parameters and safeguards should be monitored in this immunocompromised group following vaccination.

## 1. Introduction and Historical and Epidemiologic Background

When the acquired immunodeficiency syndrome (AIDS) was first described in 1981 and subsequently shown, two years later, to be caused by a previously unrecognized pathogen, human immunodeficiency virus (HIV), it was believed to be confined to a small number of risk groups that were primarily gender-based and who were of a particular sexual orientation or ethnicity [1]. As more information became known about the epidemiologic picture of HIV transmission, it became clear that the infection was transmitted primarily through sexual contact and exposure to blood either from the infusion of a contaminated blood product or through injection-drug use, as well as perinatally either from the expectant mother to the fetus or during parturition, or via breast milk [2]. With the passage of time, it eventually became clear that susceptibility to HIV infection had crept into many parts of the general population having spread beyond the originally identified risk groups. In the United States, HIV type 1 (HIV-1) is the predominant strain, whereas HIV type 2 (HIV-2) is prevalent elsewhere (e.g., in certain parts of West Africa) [3].

In 2018, approximately 38,000 new cases of HIV infection were diagnosed in the United States and its territories [3]. Although perinatal transmission in the United States has decreased to very low levels owing to routine screening for HIV and initiation of antiretroviral therapy (ART) in HIV-infected women during pregnancy, cases in adolescents and adults have decreased by only 7% between 2014 and 2018 [4]. Since the 1980s, the populations most affected by HIV infection have changed, and this affliction is now diagnosed disproportionately in persons who are impoverished, disadvantaged, and do not have regular access to routine or high-quality medical care. In 2018, 21% of new HIV infections were diagnosed in young people, 69% were diagnosed in men who have sex with men, 10% in injection-drug users, 42% in Africa-Americans, and 27% in persons of Hispanic or Latino ethnicities [3]. Around 25% of all new cases occur in white-skinned persons, who comprise about 73% of the U.S. population.

An estimated 14% of persons with HIV infection in the United States is unaware of having been exposed and of currently undergoing an active infection [3]. Many persons at greater risk—in particular large segments of racial and ethnic minorities—have limited access to skilled health care providers, and therefore, often a diagnosis is delayed until they present with advanced disease, when treatments may be less effective and the risk of death is highest [5].

In 2019, the United States began a program called the “Ending the HIV Epidemic” plan with a goal of reducing the number of new infections by 75% by 2025 and by 90% by 2030 [6]. The plan consists of four components: (i) to identify all persons with HIV infection, preferably early on in the infectious process; (ii) to successfully treat them with ART; (iii) to promote preventive measures against the development of new infections; and (iv) to initiate a quick response to unforeseen outbreaks when they occur. The basis for the first two components includes decreasing the gaps in early diagnoses, maintaining linkages to care, rapid initiation of treatment in patients with HIV infection, and maintenance of viral suppression by sustaining successful retention in patient compliance with the designated treatment regimen. It is worth noting that a more comprehensive review on the epidemiologic, clinical, diagnostic, and treatment related aspects of HIV/AIDS has recently been published [7].

## 2. Human Immunodeficiency Virus (HIV) Pathogenesis

HIV is a retrovirus that causes severe immunosuppression and leads to opportunistic infections, secondary neoplasms, and neurological abnormalities. Major transmission routes include sexual contact, parenteral transmission (IV drug users, transfusion recipients), and mother to newborn (in utero via transplacental spread, during delivery, and via breast milk).

There are two forms of HIV, HIV-1 and HIV-2, the former being the most common type in the United States, Europe, and Central Africa, and the latter present mostly in West Africa and India [7].

### 2.1. Structure of the Virus

HIV-1 has a spherical structure with an electron-dense, cone-shaped core surrounded by a lipid envelope which comes from the host cell membrane after progeny viruses emerge from an infected and now killed target cell. The virus core is made up of the major capsid protein p24 (most abundant viral antigen), the nucleocapsid protein p7/p9, two copies of viral genomic RNA, and three viral enzymes (protease, reverse transcriptase, and integrase). The viral core is surrounded by a matrix protein known as p17, lying underneath the virion envelope. The viral envelope is studded with two viral glycoproteins, gp120 and gp41, essential for HIV infection of cells [8]. The HIV-1 RNA genome contains the *gag, pol,* and *env* genes, which are common to retroviruses. The products of the *gag* and *pol* genes are large precursor proteins cleaved by the viral protease, which then results in the mature proteins being produced. HIV also contains other accessory genes, including *tat, rev, vif, nef, vpr,* and *vpu,* which regulate the synthesis and assembly of infectious viral particles and the pathogenicity of HIV [8].

### 2.2. Pathogenesis

HIV infection mainly targets the immune system, though many other tissues can be affected, including the central nervous system. AIDS, which results from HIV, causes severe immunodeficiency, mostly affecting cell-mediated immunity, via infection and death of CD4+ T cells and impairment in the function of surviving helper T cells. Infection of macrophages and dendritic cells also occurs [8]. HIV enters the body via mucosal tissues and blood and initially infects T cells as well as dendritic cells and macrophages. It becomes established in lymphoid tissues of the body and may remain latent for a long period of time, which is variable.

### 2.3. Life Cycle of HIV

The life cycle of HIV consists of infection of the aforementioned cells, integration of the provirus into the host cell genome, activation of viral replication, and production and release of infectious virus progeny. HIV infects cells via the CD4 molecule as a receptor and other chemokine receptors (coreceptors). However, this binding to CD4 is not enough for infection. HIVgp120 also needs to bind to other coreceptors for entry into the cell, especially CCR5 and CXCR4 [8,9]. Different HIV isolates are recognized by their use of these receptors: R5 strains use CCR5 and X4 strains use CXCR4. Some strains such as R5X4 use both. R5 strains are usually M-tropic, meaning they infect cells of the monocyte/macrophage lineage in addition to T cells. X4 strains are T-tropic, mostly infecting T cells. In about 90% of cases, the R5 (M-tropic) type of HIV is the dominant virus in acutely infected people’s blood, early in the infection. As the infection progresses, T-tropic viruses slowly accumulate, which are especially virulent because they can infect many T cells and even thymic T cell precursors, causing more impairment and loss of T cells [9,10].

The HIV envelope has two noncovalently associated glycoproteins, surface gp120 and gp41, the transmembrane protein. The first step in infection is binding of surface gp120 to CD4. This causes a conformational change producing a new recognition site on gp120 for the coreceptors CCR5 or CXCR4. Binding to the coreceptors causes conformational changes in gp41 so that a hydrophobic region known as the fusion peptide is exposed at the tip of gp41. This peptide inserts itself into the cell membrane of the target cells (e.g., T cells or macrophages), which leads to fusion of the virus and the host cell membrane, allowing the virus core, which contains the HIV genome, to enter the cell [9,10].

The need for HIV binding to coreceptors may be important in the pathogenesis of AIDS. Chemokines hinder HIV infection of cells in culture by occupying their receptors, and so, the chemokine levels in tissue may influence viral infection efficiency in vivo. In addition, polymorphisms in the gene encoding CCR5 are associated with different HIV infection susceptibility. About 1% of white-skinned Americans inherit two mutant copies of the *CCR5* gene and are resistant to infection and the development of AIDS associated with R5 HIV isolates. About 20% of people are heterozygous, and though not protected from AIDS, these individuals tend to progress to AIDS later on [11,12].

Once inside the host cell, the RNA genome of HIV undergoes reverse transcription, which leads to synthesis of double-stranded complementary DNA (cDNA). In dividing T cells, the cDNA circularizes, enters the nucleus, and integrates into the host genome. After integration, the provirus may be latent for months or years, or proviral DNA may be transcribed resulting in formation of complete viral particles that bud from the cell membrane. When there is extensive budding, the infected cells die [11]. HIV infects memory and activated T cells but is not efficient at productively infecting naïve (resting) T cells because these cells have an enzyme that introduces a mutation in the HIV genome. This enzyme is apolipoprotein B mRNA-editing, enzyme-catalytic, polypeptide-like 3G. It is a cytidine deaminase that introduces cytosine-to-uracil mutations in the viral DNA that are produced by reverse transcription. However, HIV has evolved to escape this defense. The viral protein Vif binds to the enzyme and causes it to degrade via cellular proteases. The viral life cycle is completed in latently infected cells only after cell activation, and for most CD4+ T cells, viral replication and shedding result in cell lysis. Activation of T cells by antigens or cytokines upregulates transcription factors, including NF-κB, that stimulate transcription of genes that encode cytokines such as IL-2 and its receptor [11]. In resting T cells, NF-κB is inactive in the cytoplasm because it is complexed with the IκB (inhibitor of κB) protein. Stimulation of cells by antigen or cytokines activates cytoplasmic kinases that phosphorylate IκB, targeting it for enzymatic degradation. This releases NF-κB and allows it to translocate to the nucleus. In the nucleus, NF-κB binds to the regulatory sequences of several genes, including those encoding for various of cytokines that are expressed in activated T cells.

The long-terminal-repeat sequences that flank the HIV genome also contain NF-κB-binding sites that can drive the expression of viral RNA. In addition, TNF and other cytokines produced by activated macrophages stimulate NF-κB activity in T cells. HIV appears to thrive when the host T cells and macrophages are physiologically activated. This activation in vivo may result from antigenic stimulation by HIV itself or by other infecting microorganisms. HIV-infected people are at increased risk for other infections, which lead to increased lymphocyte activation and production of proinflammatory cytokines. These then stimulate more HIV production, loss of additional CD4+ T cells, and more infection [11,12]. The infected CD4+ T cells are lost mostly because of the direct cytopathic effects of the replicating virus. Of note, many infected cells are within tissues (e.g., secondary lymphoid organs and mucosal sites), and death of these cells is a major cause of the extreme cell loss. As the disease progresses, renewal of CD4+ T cells cannot keep up with their loss.

Not only is there direct killing of cells by the virus, but the loss of T cells may also occur via other mechanisms. These include chronic activation of uninfected cells which leads to apoptosis of these cells so that the number of CD4+ cells that die may be much more than those infected. In addition, noncytopathic HIV infection activates the inflammasome pathway, leading to cell death via pyroptosis in which inflammatory cytokines and cell contents are released which can recruit new cells and increase the number of infected cells. HIV also infects cells in secondary lymphoid organs such as the spleen, lymph nodes, and tonsils, which may cause more destruction of the architecture and cellular make-up of lymphoid cells. Immature precursors of CD4+ T cells can also be lost either by direct infection of thymic progenitor cells or by infection of cells that secrete cytokines needed for CD4+ T cells to mature. Another way T cell loss can occur is via fusion of infected and uninfected cells with formation of giant cells. Fused cells die within a few hours. This usually occurs in the T-tropic X4 type of HIV-1, referred to as syncytia-inducing virus [11,12]. The severe reduction in CD4+ T cells can account for most of the immunodeficiency late in HIV infection. However, there is also evidence of qualitative defects in T cells even in asymptomatic HIV-infected persons. These qualitative defects include reduced antigen-induced T cell proliferation, decreased Th1-type responses, and defects in intracellular signaling, to name a few. The loss of Th1 responses results in a severe deficiency of cell-mediated immunity. This leads to increased susceptibility to infections by viruses and other primarily intracellular microbes [8].

An important feature of HIV infection is low-level chronic or latent infection of T cells. The integrated provirus, without viral gene expression (latent infection), can stay in the cells for months to years. Even with antiviral therapy, latent virus remains within the CD4+ cells (both T cells and macrophages) in the lymph nodes. About 0.05% of CD4+ T cells in the lymph nodes are latently infected, most of which are memory cells with a long life span. This provides a reservoir for the virus [12].

### 2.4. HIV Infection of Non-T Cells

Also important in the pathogenesis of HIV infections is infection of macrophages and dendritic cells. Just like in T cells, the number of HIV infected macrophages in tissues is much greater than the number of infected monocytes in the blood. Some organs like the lungs and brains may have up to 10% to 50% of infected macrophages [12]. HIV-1 can infect and multiply in terminally differentiated nondividing macrophages. This depends on the viral *vpr* gene. The Vpr protein permits nuclear targeting of the HIV pre-integration complex via the nuclear pore.

Infected macrophages bud small amounts of virus from the cell surface but contain a large amount of virus particles, often in intracellular vesicles. Unlike CD4+ T cells, macrophages are resistant to the cytopathic effects of HIV. Therefore, in late-stage HIV infection when CD4+ T cells are greatly depleted, macrophages may be an important site of continued viral replication and a viral reservoir. The reason why macrophages may be important portals of infection is because, in more than 90% of cases, acute HIV infection is caused by M-tropic strains [12]. Uninfected monocytes may also have unexplained functional defects including impaired microbicidal activity, decreased chemotaxis, decreased secretion of IL-1, inappropriate secretion of TNF, and poor processing capability needed to present antigens to T cells. In addition, infected monocytes may carry the virus from the blood to other parts of the body, such as the nervous system, where it can infect microglial cells. Two types of dendritic cells are also important in HIV infection: mucosal and follicular dendritic cells. It is believed that mucosal dendritic cells are infected by HIV and may transport it to regional lymph nodes, where HIV is transmitted to CD4+ T cells. In addition, dendritic cells also have a lectin-like receptor that binds HIV and displays it in an intact, infectious form to T cells, which promotes infection of T cells. Follicular dendritic cells in the germinal centers of lymph nodes are potential reservoirs of HIV. Most virus particles are present on the surface of their dendritic processes. Follicular dendritic cells have receptors for the Fc portion of immunoglobulins. They can then trap HIV virions coated with anti-HIV antibodies. These antibody-coated virions on the follicular dendritic cells have the ability to infect CD4+ T cells. Infected T follicular helper cells in the germinal centers are also reservoirs of HIV [12]. Because CD4+ T cells are needed in regulating both cellular and humoral immune responses, this loss greatly affects the immune system.

### 2.5. HIV and B Cells

Although HIV infects T cells, macrophages, and dendritic cells, people with AIDs also have abnormalities in B-cell function. Early in the disease process, there is polyclonal activation of B cells, causing germinal center B-cell hyperplasia. B-cell activation may occur via reactivation of, or reinfection with, Epstein Barr virus, which is a polyclonal B-cell activator; viral gp41 (which can promote B-cell growth and differentiation); and increased IL-6 production, which stimulates proliferation of B cells, by HIV-infected macrophages [9,12]. Despite the increased activation of B cells and risk of autoimmunity, patients with AIDS cannot produce effective antibody responses. This may partly be because of lack of T-cell help but apparently antibody responses against T-independent antigens are also suppressed, so there may be intrinsic B cell defects. Furthermore, this impaired humoral immunity makes patients susceptible to disseminated infections caused by encapsulated bacteria (e.g., *Streptococcus pneumoniae* and *Haemophilus influenzae)*, which need antibodies for opsonization and clearance [8].

### 2.6. HIV and the Nervous System

The nervous system is also affected by HIV infection. Microglia in the CNS are the main cell types infected with HIV. It is thought that infected T cells or monocytes carry HIV into the brain though it is not certain how HIV induces damage. Because neurons are not infected by HIV and neuropathologic changes are less than expected considering the severity of neurologic symptoms, it is thought that the neurologic deficit is caused indirectly by viral products and by soluble factors produced by infected microglia (e.g., IL-1, TNF, and IL-6). It is also thought that gp41 induces nitric oxide in neurons and that direct damage of neurons by soluble HIV gp120 may occur [9,12].

### 2.7. HIV and Renal Disease

Another organ affected by HIV infection is the kidney. HIV-associated nephropathy (HIVAN) is a leading cause of end stage renal disease in HIV-1 positive patients and may lead to death [13,14]. Classically, the clinical presentation of HIVAN involves progressive azotemia, rapidly progressive renal failure, proteinuria ranging from moderate to nephrotic range (though usually little to no peripheral edema), bland urinary sediment, and large, highly echogenic kidneys based on ultrasound results [13,14].

In a study of 107 HIV infected patients who underwent kidney biopsy between 1995 and 2002, those who had classic HIVAN were also more likely to have a CD4 cell count <200 cells/mm^3^ [14]. In autopsy studies, gross examination has revealed enlarged, pale, swollen kidneys in patients with HIVAN [14]. Microscopically, there is renal parenchymal injury showing epithelial cell proliferation, dedifferentiation, and apoptosis along the nephron, with collapsing focal segmental glomerulosclerosis (FSGS), microcystic tubular dilation, interstitial inflammation, and fibrosis [13]. Untreated HIVAN patients in the acute phase usually show a dramatic pattern of collapsing FSGS with glomerular capillary lumina occluded by wrinkling and collapse of the glomerular basement membrane [14]. This is associated with prominent hypertrophy and hyperplasia of the overlying podocytes, showing enlarged, open vesicular nuclei with frequent nucleoli, occasional binucleate forms, and rare mitotic figures [14]. Pseudocrescents may form because of the very crowded visceral epithelial cells that obliterate the urinary space. The cytoplasm of the podocytes is usually vacuolated with prominent intracytoplasmic protein resorption (hyaline) droplets [14]. Over time, the glomerular tuft retracts into a tight, solid ball crowned by overlying enlarged, vacuolated visceral epithelial cells.

### 2.8. HIV Infection Natural History

Acute (early) HIV infection is marked by infection of memory CD4+ T cells (which express CCR5) in mucosal lymphoid tissues and leads to death of many infected cells. Because the mucosal tissues are the largest reservoir of memory T cells in the body and memory T cells are susceptible to HIV infection, this results in a great loss of lymphocytes. Mucosal infection is often associated with damage to the epithelium, defects in mucosal barrier functions, and translocation of other microbes across the epithelium [9]. After mucosal infection, dissemination of the virus and development of host immune responses occur. Dendritic cells in epithelia at virus entry sites capture the virus and then migrate into the lymph nodes. Once there, the dendritic cells may pass HIV on to CD4+ T cells via direct cell-to-cell contact. Viral replication can be detected in the lymph nodes within days after the first exposure to HIV. This leads to viremia, so that high numbers of HIV particles are present in the patient’s blood. The virus disseminates throughout the body and infects helper T cells, macrophages, and dendritic cells in peripheral lymphoid tissues [9].

As the HIV infection spreads, antiviral humoral and cell-mediated immune responses are produced by the patient. These lead to seroconversion (usually within 3 to 7 weeks of exposure) and the development of virus-specific CD8+ cytotoxic T cells—one of the remaining non-humoral immune system components not directly affected by the virus. HIV-specific CD8+ T cells are detected in the blood around the same time viral titers begin to fall and are most likely the cause of initial containment of HIV infection. These immune responses partially control the infection and viral production. This is seen by a drop in viremia to low but detectable levels by about 12 weeks after primary exposure [9]. The “acute retroviral syndrome” clinically reflects the initial spread of the virus and the host response. About 40 to 90% of people who acquire a primary infection develop this syndrome, which usually occurs 3 to 6 weeks after infection and resolves spontaneously in the next 2 to 4 weeks. It is characterized by a nonspecific self-limited acute illness with flu-like symptoms, such as sore throat, myalgias, fever, weight loss, and fatigue, sometimes with a rash, cervical adenopathy, diarrhea, and vomiting [11].

Measuring the viral load or level of HIV-1 RNA in the blood is a useful way to monitor HIV disease progression (refer to diagnosis below) and is helpful for clinical management, especially in the course of the patient receiving antiretroviral treatment. However, the blood CD4+ T-cell count is probably the most reliable short-term indicator of disease progression. Therefore, the CD4+ cell count (rather than viral load) is the primary clinical measurement used to determine when to start antiretroviral therapy (ART) [7].

### 2.9. Chronic HIV Infection

In the chronic phase of HIV infection, the areas of continuous HIV replication and cell destruction are the lymph nodes and spleen. During this period, there are few or no clinical manifestations of the HIV infection, and this is known as the clinical latency period. Although the majority of peripheral blood T cells do not have the virus, destruction of CD4+ T cells within lymphoid tissues continues and the number of CD4+ T cells in the blood continues to decline. Over a period of years, the slowly amplifying cycle of virus infection, T cell death, and new infection results in a continued decline in CD4+ T cell numbers in the lymphoid tissues and peripheral circulation [9]. Along with this loss of CD4+ T cells, host defenses decline, and the proportion of surviving CD4+ cells infected with HIV increases, as well as the viral burden per CD4+ cell. HIV RNA levels increase. Although not completely elucidated, HIV may escape immune control because of destruction of the CD4+ T cells needed for effective immunity, antigenic variation, and down-modulation of class I MHC molecules on infected cells so that viral antigens are not recognized by CD8+ CTLs. During this period, the virus may evolve and switch from only relying on CCR5 to enter its target cells to relying on either CXCR4 or both CCR5 and CXCR4. This coreceptor switch is associated with more rapid decline in CD4+ T-cell counts, because more T cells are infected. Patients in this chronic phase of infection are either asymptomatic or develop certain types of opportunistic infections, such as oral candidiasis (thrush), vaginal candidiasis, herpes zoster, and tuberculosis [8].

### 2.10. AIDS

The final phase of HIV infection is the development of AIDS with a breakdown of host defenses, a great viral load increase, and profound, life-threatening clinical disease. The typical person with AIDS presents with long-lasting fever (>1 month), fatigue, weight loss, diarrhea, and generalized lymph node enlargement. After a period, which varies, a wide range of serious opportunistic infections, secondary neoplasms, or clinical neurologic disease may develop [7,8]. Without treatment, most patients with HIV infection progress to AIDS after a chronic phase lasting from 7 to 10 years. However, some patients are rapid progressors or long-term nonprogressors. In the former group, the middle, chronic phase is 2 to 3 years after primary infection. About 5 to 15% of infected individuals are long-term nonprogressors, defined as untreated HIV-1-infected individuals who remain asymptomatic for 10 years or more, with stable CD4+ T-cell counts and low viral loads. There are even about 1% of infected individuals (elite controllers) who have undetectable plasma virus [12]. A typical adult patient in the United States with AIDS presents with fever, weight loss, diarrhea, generalized lymphadenopathy, multiple opportunistic infections, neurologic disease, and, in many cases, secondary neoplasms. Collectively, these clinical abnormalities include pneumocystis pneumonia, toxoplasmal encephalitis, candidiasis, cryptococcal meningitis, atypical mycobacterial infections, cytomegalovirus infection, Kaposi sarcoma, primary lymphoma of the brain, and progressive multifocal leukoencephalopathy [8,12]. Ninety percent of patients show some form of neurologic involvement at autopsy, and 40% to 60% have clinical neurologic dysfunction. With treatment, the mortality rate in the U.S. has declined, over the past several years, but treated patients still carry viral DNA in their lymphoid tissues. Molecular analyses show a lot of variation in viral isolates from patients, which makes vaccine development difficult as a preventive measure against HIV infection. There have been some recent efforts focusing on producing broadly neutralizing antibodies against relatively invariant portions of HIV proteins [9].

### 2.11. Diagnosis of HIV/AIDS

**Figure 1:**
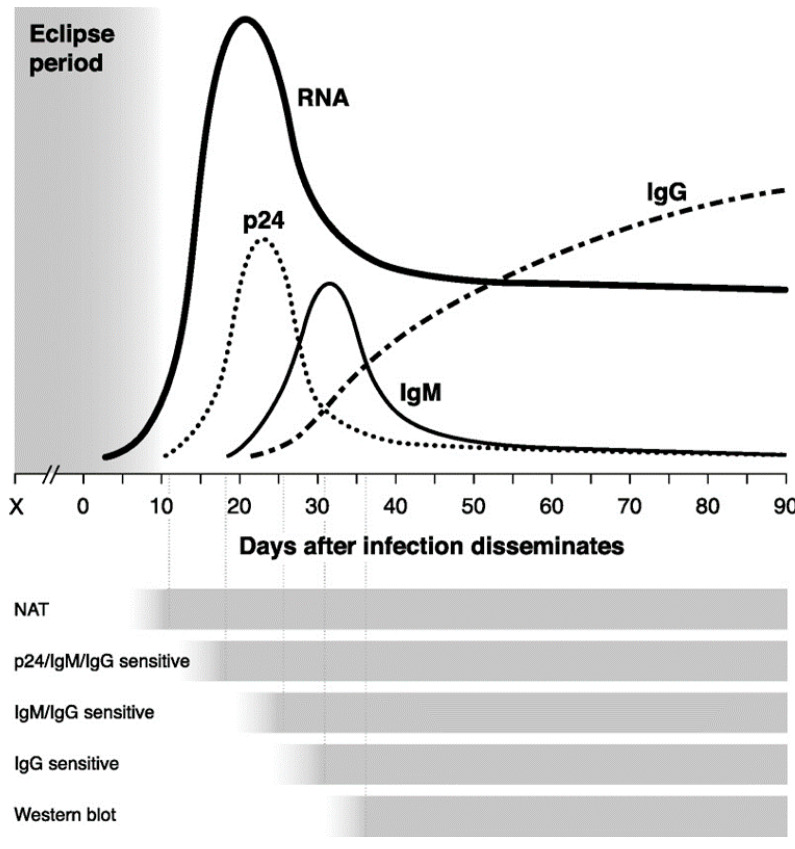
Timeline of virologic and serologic events associated with HIV infection. The length of time between when exposure to the virus occurs and there is dissemination of HIV is systemically dependent upon the manner in which the virus is acquired. The eclipse period represents the time from the exposure event to the first detectable marker of infection—when HIV RNA appears in the blood. Times to reactivity for each type of diagnostic test are depicted under the graph, from the earliest one—the nucleic acid amplification test (NAT)—to the latest assay system (test for IgG antibodies). A Western blot (or immunoblot) is used to confirm an initial positive result from a standard serologic test such as an ELISA. This latter aspect of the serologic algorithm is to ensure that an initial serologically-derived positive test result is not a false-positive. A Western blot is also not supposed to be used initially as a screening test, irrespective of the patient presentation, primarily for cost-containment purposes. Adapted from Maag, 2021 [7].

Apart from the clinical presentation and an accurate medical history, several tests are available to accurately diagnose or confirm an HIV infection (summarized in Figure 1). The choice of the most appropriate test for a given clinical presentation depends on an understanding of the natural history of HIV infection (i.e., which marker is present at a given point after infection, as shown in Figure 1), the volume of the specimen, and the test-performance specifications [7]. Another, perhaps an underappreciated consideration, would be the testing capabilities of the infrastructure of the local health care environment. During the “eclipse” period, before viremia becomes established at day 5, infection cannot be detected. By days 6 to 8, virus can be detected by a nucleic acid amplification test, such as the polymerase chain reaction (PCR). Viral proteins, such as the p24 antigen, can be detected between days 13 and 20. Antibodies, initially in the form of IgM, are detectable by day 20, and IgG is detectable by day 30 [7]. Many patients seek medical care long after the initial infection, when tests for antibodies and viral RNA are both positive [7].

## 3. COVID-19 Pathogenesis

COVID-19 is caused by a unique virus called SARS-CoV-2. It belongs to the Coronoviridae family of viruses [15] displaying a crown-like morphology with spike (S) glycoproteins radiating from its surface [16]. All the coronaviruses are large, enveloped, single-stranded RNA viruses that are found in humans and animals [17]. SARS-CoV-2 major structural proteins include a spike surface glycoprotein (S), a small envelope protein (E), a matrix protein (M), and a nucleocapsid protein (N) [15]. The N protein forms as a helical capsid to pack the genome, which is wrapped with an envelope consisting of the spike surface glycoprotein (S), a small envelope protein (E), and a matrix protein (M) [15]. The S glycoprotein helps regulate binding of the receptors and the virus into host cells, and the E protein and M protein are involved in virus assembly [13]. The S protein has three segments—an ectodomain, a single-pass transmembrane anchor, and a short intracellular tail. The ectodomain has a receptor-binding S1 domain and a membrane-fusion S2 domain, which are very important for virus entrance into the host cells via receptor binding and membrane fusion. The envelope is also important because it is involved in viral assembly and the V-release process [15].

SARS-CoV-2, like other RNA viruses, has a great deal of genetic variability because there is a lack of proof-reading activities of viral RNA-dependent polymerase, making it more adaptable to survival [18]. There is variability of the amino acid sequences in the spike protein for host cell receptor binding and the virus has the capability to evolve to be more replication efficient. In addition, some of the viral proteins may evolve into mediators that do not allow the host immune system to recognize or attack infected cells. The virus may also be able to develop drug resistance [18].

COVID-19 is most commonly transmitted via contact with respiratory droplets from talking, coughing, and sneezing during face-to-face exposure [15]. Direct inhalation of infected particles and contact transmission via oral, nasal, and eye mucous are also important. The incubation period of the virus is thought to be usually 3–7 days but can be up to two weeks, although 97.5% of individuals who do develop symptoms do so within 11.5 days of infection [17]. After being inhaled, SARS-CoV-2 enters the host cells first by binding of the spike protein to the angiotensin converting enzyme (ACE)2 receptor on the cells’ surfaces [16]. This receptor is found on many types of tissues in the body including the lungs (especially type 2 pneumocytes in the alveoli), blood vessels, heart, liver, kidneys, upper respiratory tract epithelium, and the gastrointestinal tract [16]. This helps explain the frequency of pneumonia as well as vasculitis, the presence of fecal viral RNA and antigen detection, as well as other organ disease manifestations [18]. After the binding of virus to the ACE2 receptor on the host cell and subsequent entry into the cell, the virus replicates, and in later stages, the viral load becomes higher causing compromise of the epithelial-endothelial barrier [15]. Of note, smokers have an increased lung expression of ACE2 receptors [18], which could exacerbate disease progression.

The host response includes elicited inflammation early on, which results in an infiltration of numerous monocytes and neutrophils to the target sites [17]. In addition, inflammatory signaling molecules are released by infected cells, alveolar macrophages and recruited monocytes, neutrophils, and T lymphocytes [18]. As the disease progresses, SARS-CoV-2 virus infects pulmonary capillary endothelial cells, which also triggers an influx of monocytes and neutrophils, killing T lymphocyte cells, further increasing the inflammatory response. This results in a thickened interstitium, hyaline membrane formation, pulmonary edema, and activation of coagulation factors, which can lead to microthrombi [16]. Viral sepsis may develop, which further leads to multiorgan failure [18]. Therefore, it appears that the viral infection causes an excessive immune response, with the key aspect being what has come to be known as a “cytokine storm”—leading to critical illness and death due to severe pneumonia and other systemic complications [17]. The interstitial mononuclear inflammation and edema which develop in the lung are seen by computed tomographic imaging as ground glass opacities [19]. The pulmonary findings can be divided into earlier and later phases with earlier manifestations of pulmonary edema, protein exudation, vascular congestion, pneumocyte hyperplasia, interstitial thickening, inflammation with fibrinoid material and multinucleated giant cells, and hyaline membrane formation [15]. Later features include diffuse alveolar damage with fibrous mucus-like exudates, desquamation of pneumocytes, and hyaline membrane formation (as found in acute respiratory distress syndrome) [15].

The first completed autopsy report of a deceased COVID-19 patient was released in February 2020 and showed an extensive inflammatory reaction with deep airway and alveolar damage [18]. Electron microscopy examination of autopsy specimens showed large numbers of viral particles in alveolar epithelial cells [20]. Other postmortem studies have shown that the lungs, especially the middle and lower lung lobes, were adherent to the chest wall, implying inflammation of the peripheral lung tissue leading to the formation of adhesions. Microscopically, the findings included the presence of diffuse alveolar wall thickening with mononuclear cells and macrophages infiltrating the alveoli and endothelialitis. Many patients also had secondary bacterial infections, while a few had secondary fungal infections [18]. Other key postmortem findings from different studies have included the presence of large amounts of chemokines coming from the macrophages in the bronchoalveolar fluid (in severe disease), damage to the alveoli with interalveolar hemorrhage, vascular congestion, and type 2 pneumocyte hyperplasia [19]. In addition, myocarditis and cardiomyopathy, fibrin thrombi in alveolar arterioles, and microthrombi (indicating coagulation problems) in the lungs, liver, brain, heart, lower limbs, hands, and kidneys have been described. Neurological postmortem findings included hemorrhagic white matter lesions throughout the cerebral hemispheres, axonal injury, clusters of macrophages, and a perivascular acute disseminated encephalomyelitis-like appearance [19]. A more recent systematic review and meta-analysis of postmortem findings that are associated with COVID-19 was recently published [20], which showed that the most common cause of mortality was diffuse alveolar damage. However, there were also other remarkable findings, which included thromboembolism, brain infarction, endotheliitis, acute renal tubular damage, white pulp depletion in the spleen, necrosis of cardiac myocytes, recruitment of megakaryocytes, and hemophagocytosis [20].

### Diagnosis of COVID-19

As soon as the COVID-19 pandemic began to emerge, the default “gold standard” test, which diagnostic laboratories have relied on, and continue to rely on, was a nucleic acid amplification test, most notably PCR, to detect people who have been infected with SARS-CoV-2 [21,22,23]. As with most difficult-to-culture pathogens, PCR has replaced the cumbersome and time-consuming in vitro culture of the virus from patient samples for diagnostic purposes, although some versions may require several hours before results are available, often depending upon the workload of the testing facility. The sensitivity and specificity of this assay system is relatively high and assures quick and reliable identification of infected persons who may go on to develop serious disease and potentially transmit the virus unwittingly to others [22,23].

The next most reliable assay system for detecting COVID-19 is serology. The detection of anti-SARS-CoV-2 antibodies is useful when PCR is not available for confirming suspicious cases, or when PCR test results are repeatedly negative yet there is a high suspicion of disease, or the patient presents late in disease when viral RNA may be very low or absent from a patient sample, such as a nasal swab or blood. Owing to the high level of serology’s sensitivity, antibodies appear relatively early (2–3 weeks of infection) [21] and are directed against the spike and nucleocapsid antigens. An additional benefit of serology is the identification of prospective donors of convalescent plasma for use in certain treatment regimens [24].

Unfortunately, in many countries, especially those with weak health care systems or infrastructure and/or limited laboratory capabilities, access to these two forms of testing can be challenging and/or it is difficult to get timely results. As an alternative, antigen-detection systems have been developed (reviewed in Reference [25]) which could alleviate this problem. These tests are intended for the qualitative detection of key antigens, such as the nucleocapsid protein, from SARS-CoV-2 in nasal swabs, from people suspected of having contracted COVID-19 by their health-care provider, and similarly to PCR, such results can be obtained within the first few hours or less of symptom onset. Patient samples are tested immediately after being collected, and there is no need in some platforms to dilute the sample in any type of transport media or solution prior to applying the sample onto the test device. These devices are relatively inexpensive and compact (about the size of a credit card), can be purchased at most local pharmacies without a prescription, can be performed outside the confines of a health care facility, and are capable of providing results in approximately 15–30 min. In most cases, results can be read visually without the need for any additional equipment or instrumentation. Unfortunately, while the sensitivity of most of the currently available antigen-detection tests is excellent, their sensitivity has been shown to vary from low to high [25]. A comparison of the various features, including the performance characteristics of the three different detection systems, is shown below in Table 1.

## 4. COVID-19 Vaccines for the HIV-Infected Patient

The speed with which safe and effective vaccines have been developed to prevent COVID-19 has been nothing short of remarkable and perhaps even spectacular. In just a few months after the pandemic became recognized as a global nightmare and a sense of urgency had crept into the picture, potential vaccines were produced by several manufacturers and became available for testing. This outcome is a testament to the hard work and diligence of the research community and pharmaceutical industry in cooperating in this venture, along with the huge response of the general population willing to participate in clinical trials that were needed to verify the worthiness of these vaccines. Even many of the recognized medical/scientific “experts” had predicted that vaccines would likely not be available for routine use for almost two years after the initiation of experiments to try to develop them. Certainly, in the back of many minds was the gloomy feeling that it may even take much longer given the relative lack of success, after much effort and a large amount of funds had already been invested, over a span of many years, in trying to develop effective vaccines for malaria, HIV infection, and EBOLA virus disease (EVD)—diseases having high rates of morbidity and mortality (similar outcomes to COVID-19), but which have been known and well characterized for well over a century (for malaria) and for nearly a half century (for HIV infection and EVD).

As of August 2021, two types of anti-COVID-19 vaccines are in use and available to everyone from ages twelve and above. One type consists of purified messenger RNA (mRNA) given in two intramuscular injections with the second injection given 3–4 weeks after the first one. The mRNA encodes for the SARS-CoV-2 spike protein. The other vaccine has a viral vector formulation with a live but safe adenovirus serving as the vector for carrying the genome of the SARS-CoV-2 spike protein and only needs to be given once in one of the two versions currently available (Table 2). In both cases, the spike protein had been shown to be highly immunogenic and the key protective component in pre-clinical studies and is associated with a vigorous antibody response. After an initial Emergency Use Authorization (EUA) that was issued in December 2020 and then followed by a second one in February 2021 by the U.S. Food and Drug Administration (FDA), the two mRNA vaccines produced by Pfizer (Pearl River, NY, USA) and Moderna (Cambridge, MA, USA) are pending final approval for routine use as of August 2021. This is also true for the viral vector vaccines produced by Janssen/Johnson & Johnson (Titusville, NJ) and Astra-Zeneca (Cambridge, UK), which have received an EUA for adults (≥15 year’s old) in the U.S. It should be noted that in the United Kingdom much earlier approval was given in December 2020 and January 2021, for some of these vaccines, by the U.K. Health Department following recommendations by the Medicare and Health Care Regulatory Authority in the Department of Health and Social Care. The key features of these four vaccines are summarized in Table 2. It is worth noting that, from the very beginning, the rapid deployment of these vaccines, especially in the U.S., was partially aided by the involvement of the U.S. federal government under the program known as “Operation Warp Speed” [26]. Although some people may have viewed this program as being partially politically motivated, it provided considerable financial and logistical support to the pharmaceutical industry for accelerating the development and distribution of multiple vaccines throughout much of the U.S. in a nearly unprecedented fashion.

As with the general population, people with HIV/AIDS should receive one of the available vaccines as a preventive measure against developing COVID-19. Interestingly, in the overall picture, the evidence to date (reviewed in Reference [27]) does not suggest that HIV/AIDS patients have a markedly higher susceptibility to SARS-CoV-2 infection, with disparities in the social determinants of health and comorbidities likely having a greater influence, especially with regards to gaining access to supportive health care and continuation of ART. There are also other reports from separate facilities describing increased, decreased, or no difference in outcomes of COVID-19 in this patient population, especially in terms of the fatality rate [26]. These studies have come from various locations, each with a different underlying HIV prevalence and access to various ART regimens. The majority of the published literature has not supported a significantly higher risk for severe disease among HIV/AIDS patients in the United States and Europe, although a large, population-based study in South Africa reported a higher rate of death due to COVID-19 [28]. Higher rates of comorbidities associated with COVID-19 disease severity among HIV/AIDS patients is an area that still needs to be monitored closely. The immediate impact of COVID-19 is that it could lead to decreased access to HIV prevention services and HIV testing, and hampering HIV treatment access and virologic suppression could lead to worsening of HIV control and other desirable positive outcomes. In addition, along these lines, the U.S. Centers for Disease Control and Prevention (CDC) has concluded [29] that people with HIV who are on effective HIV treatment have the same risk for developing COVID-19 as people who do not have HIV, although the risk for people with HIV getting very sick is greatest for those who have a low CD4 T-cell count and are not on effective HIV treatment such as with ART.

Based on the foregoing, a few relevant questions arise as follows: (i) Should HIV/AIDS patients receive a COVID-19 vaccine? (ii) Are the vaccines safe for this patient population? (iii) If so, which vaccine should be administered to them? (iv) How often should they receive booster injections? (v) At what point, if at all, should they be vaccinated after they have recovered from an infection with SARS-CoV-2? The answers to most of these questions are relatively straightforward and not complicated. According to the CDC [29], the U.S. vaccine safety system makes sure all vaccines are as safe as possible. COVID-19 vaccines have gone through rigorous safety tests and have met or even exceeded similar standards to those for other vaccines that have been produced for nearly a century and have been in routine use for over 60 years. People with HIV have been included in clinical trials, though there is limited safety and efficacy data available as they pertain specifically to this group. So far, there are no data to suggest that the vaccines are not safe and effective for people with HIV, including adolescents between 12 and 15 years, nor has it been shown that the COVID-19 vaccines interfere with the effectiveness of HIV medications such as ART. There have been no unusual links or enhanced negative reactions between HIV or other types of immunosuppression with any of the rare serious adverse events that have been reported for the COVID-19 vaccines. Much of this information has been provided by the British HIV Association [30] and the World Health Organization [31], who have also indicated that HIV-infected people generally will likely produce a weaker response to the COVID-19 vaccines, but they are still expected to be protective. This protection, however, may be to a lesser extent, especially for those individuals with low CD4+ counts (less than 100). Nonetheless, the U.K. Department of Health recommends that people with HIV, regardless of their CD4+ count, should receive a COVID-19 vaccine. In addition, because some people with HIV, especially those with a very low CD4 T-cell count, may be at increased risk for severe illness due to COVID-19, the CDC recommendation [29] advises that people with HIV may receive the vaccine as long as they do not have other conditions that would exclude them, such as a known severe allergic reaction or immediate allergic reaction of any severity after a previous dose or to a component of the COVID-19 vaccine. The vaccines authorized for use in the United States and the United Kingdom do not contain infectious virus so they are expected to be safe in people with low CD4 cell counts. It is worth noting, however, that while the currently available vaccine products are not live vaccines, in the traditional sense, the one produced by Jannsen/Johnson & Johnson and Astra-Zeneca uses a live, but replication-incompetent human adenovirus vector, encoding for the recombinant SARS-CoV-2 spike (S) glycoprotein, stabilized in its pre-fusion form. The viral vector’s purpose is to introduce the DNA, which encodes for the spike protein, into the human body in a somewhat unique way so that multiple copies of it can be produced in vivo, thus making a repeat (i.e., a booster) injection theoretically less likely to be necessary. Such a viral vector vaccine is considered to be safe even for immunocompromised patients, including those with HIV/AIDS, given that the viral vector has been shown to be harmless in one of the most recently published studies on this topic [32]. Nonetheless, prior to this finding, there was some initial concern about a potential association observed more than a decade ago between adenovirus vector-based vaccines and an increased risk of acquiring HIV infection among men who received this type of vaccine [33]. This unexpected finding was detected in two HIV vaccine trials that used adenovirus vector-containing products [34,35], but these vaccines were constructed differently and are not related to the structure of the COVID-19 vaccines. The reason for this observed HIV risk remains uncertain, although several follow-up studies have suggested a possible interference in the HIV-specific vaccine response or in the CD4 cell susceptibility to HIV infection induced by this kind of vaccine [36,37]. Accordingly, specific studies on this issue with this type of COVID-19 vaccine should be considered by closely monitoring the response patterns of HIV-infected people to various immune parameters, which would include periodically measuring CD4 T-cell counts, viral loads, and anti-spike protein antibody levels subsequent to being vaccinated. In addition, testing for delayed-type hypersensitivity responses, as shown recently by Barrios et al. [38] for recovering COVID-19 patients, may also provide valuable insights on the importance of cellular immune responses mediated by CD8+ T cells that directly kill virally infected cells as an additional defense mechanism for prospective vaccinees. There are still, however, other unresolved issues. For example, will the currently available vaccines be fully protective against variants of SARS-CoV-2, especially the more recently identified highly invasive/infectious delta variant [39,40], what is the longevity of protection that is provided by any of these vaccines, and will additional boosters be needed beyond what is currently being done? Somewhat encouraging news, along these lines, was recently reported [41] indicating that the Janssen/Johnson & Johnson vaccine provided lasting protection of at least 8 months duration and it afforded protection against the delta and other variants. Although there had already been prior preliminary evidence (reviewed in Reference [42]) supporting an important role for both CD4+ and CD8+ T cells in the immunologic memory component in the host response to COVID-19, it is likely that additional related news on these topics will be forthcoming in the coming months. In light of these potential concerns/issues, it should be realized that, in the final analysis, the overall benefits of receiving any of the authorized COVID-19 vaccines in a pandemic situation currently outweigh the potential risks, even for people with impaired immune systems.

## Figures and Tables

**Table 1:** Summary of some of the similarities and differences between molecular-based tests (for example, RT-PCR), serologic assays and antigen-detection tests that are currently in use for the diagnosis of COVID-19.

	RT-PCR Tests	Serologic Assays	Antigen Tests
Intended Use	Detect current infection	Detect current/past infection	Detect current infection
Type of Analyte Detected	Viral RNA	Immunoglobulin(s)	Viral antigens
Specimen Types(s)	Nasal swab; Saliva	Serum or plasma	Nasal swab; saliva
Sensitivity	High	Moderate to high	Low to moderately high
Sensitivity	High	Moderate to high	High
Test Complexity	Variable	Variable	Relatively easy to use
Authorized for use at the point-of-care site	Most formats are not, some formats are allowed	Same as PCR	Yes
Turnaround time for a test result	Ranges from about 15–30 min to >2 days	Same as PCR	About 15–30 min
Cost per test	Moderate	Moderate	Low
Screening	No	No	Yes
Confirmation	Yes	Yes	No or yes
Persistence of analyte after recovery	No	Yes ^a^	No

^a^ Detectable level of antibodies tend to decrease gradually over time with the major isotype being IgG. Adapted from Pavia and Plummer [25].

**Table 2:** Comparison of the key features of the COVID-19 vaccines that are produced by the four leading manufacturers.

Vaccine Manufacturer.	Type of Vaccine	Number of Doses	Authorized for Use in the U.S. ^a^ Pending Final Approval	Emergency Use Authorization ^b^	Serious Adverse Events	Percent Efficacy
Moderna	mRNA	2	yes	yes ^c^	rare	>94%
Pfizer	mRNA	2	yes	yes ^c^	rare	>94%
Janssen	viral vector	1	yes	yes	yes ^d^	<90%
Astra-Zeneca	viral vector	2	yes	yes ^c^	yes ^d^	<90%

^a^ Information that was available as of August 2021. ^b^ In the U.S., an EUA has been given for these vaccines for people aged ≥16, which was subsequently expanded to ≥12 years of age. ^c^ In the U.K., an EUA no longer applies for the Pfizer, Moderna, and Astra-Zeneca vaccines, which now have been granted final approval for use. In the U.S. as of August 2021, the Pfizer vaccine was given FDA approval, while the other 3 vaccines are undergoing further evaluation for full approval by the FDA. ^d^ Blood clots have been reported in a small number of mostly female vaccine recipients ≤50 years of age or ≥65 of age for these two vaccines. Myocarditis and pericarditis have occurred rarely in people ≤30 years of age who received the Moderna or Pfizer vaccine.
